# Effect of Vibrotherapy on Body Fatness, Blood Parameters and Fibrinogen Concentration in Elderly Men

**DOI:** 10.3390/jcm10153259

**Published:** 2021-07-23

**Authors:** Anna Kabata-Piżuch, Agnieszka Suder, Paweł Jagielski, Katarzyna Kubasiak, Paulina Handzlik, Aneta Teległów, Anna Marchewka

**Affiliations:** 1 Department of Anatomy, Faculty of Physical Rehabilitation, University of Physical Education, 31-571 Krakow, Poland; DT-417@student.awf.krakow.pl (A.K.-P.); katarzyna.kubasiak@awf.krakow.pl (K.K.); paulina.handzlik@interia.pl (P.H.); 2 Department of Nutrition and Drug Research, Faculty of Health Science, Jagiellonian University Medical College, 31-066 Krakow, Poland; paweljan.jagielski@uj.edu.pl; 3 Department of Clinical Rehabilitation, Faculty of Physical Rehabilitation, University of Physical Education, 31-571 Krakow, Poland; aneta.teleglow@awf.krakow.pl (A.T.); anna.marchewka@awf.krakow.pl (A.M.)

**Keywords:** body mass index, body fat, blood parameters, fibrinogen, physiotherapy

## Abstract

Elderly people need activities that will positively contribute to a satisfactory process of getting older. Vibration training uses mechanical stimulus of a vibrational character that, similarly to other forms of physical activity, affects metabolic processes and conditions of health. The aim of this work was to assess the influence of thirty vibration treatments on body fatness, hematologic and rheologic indexes of blood, and proteinogram and fibrinogen concentration in elderly men’s blood. The study included twenty-one males, aged 60–70 years (mean age 65.3 ± 2.7), who were randomly assigned into a vibrotherapy group (VG) and took part in interventions on mattresses generating oscillatory-cycloid vibrations, and a control group (CG), without interventions. In all patients the following assessments were performed twice: an assessment of body fatness using the bioimpedance method, a complete blood count with a hematology analyzer, and erythrocyte aggregation by a laser-optical rotational cell analyzer; whereas, total plasma protein and fibrinogen values were established, respectively, by biuret and spectrophotometric methods. In order to compare the impact of vibrotherapy on changes in the analyzed variables, analysis of variance (ANOVA) or the Wilcoxon test were used. After applying thirty vibration treatments in the VG, a significant decrease in body fatness parameters was confirmed: BM (∆BM: −2.7 ± 2.0; *p* = 0.002), BMI (∆BMI: −0.9 ± 0.7; *p* = 0.002), BF (∆BF: −2.5 ± 2.5; *p* = 0.013), and %BF (∆%BF: −2.0 ± 2.7; *p* = 0.041), as well as in RBC (∆RBC: −0.1 ± 0.1; *p* = 0.035). However, changes in erythrocyte aggregation and proteinogram were not confirmed. It was found that after thirty treatments with VG, a significant decrease of fibrinogen level took place (∆ = −0.3 ± 0.3, *p* = 0.005). Application of thirty vibrotherapy treatments positively affected body fatness parameters and fibrinogen concentrations in the examined. However, further research should include a greater number of participants.

## 1. Introduction

The positive effects of physical activity for lowering the risk of many diseases and the improvement of life quality in elderly people are well known and documented [1]. Vibration training is one of the physiotherapy methods that uses mechanical stimulus of a vibrational character. It can affect a selected group of movement organs or the whole body (WBV). The main parameters of vibration include frequency: number of oscillations per minute within a range from 0.5 Hz to 100 Hz, amplitude: maximal inclination from the state of equilibrium in oscillation motion expressed in mm (from 0.01 mm to 5 mm), and acceleration: defined as maximal change of velocity in a oscillation cycle given in m/s^2^ (from 0.01 m/s^2^ to 10 m/s^2^). Vibrotherapy applies technology of a system of controlled therapeutical oscillations, which allow the transfer of a vibrational stimulus of an oscillatory-cycloidal character and generating mechanical waves spreading simultaneously in three directions, thus increasing their permeability [2]. Vibration activates muscle spindles, causing tonic vibration reflex (TVR) relaxation at the same time as their main antagonists [3]. Therefore, the vibrational stimulus can, similarly to other forms of exercise, influence metabolic processes and conditions of health [4]. Elderly people are frequently affected by the negative effects of bedrest confinement, and vibration therapy applied in a lying position seems to be an efficient means of combating the deconditioning or to prevent orthostatic intolerance [5,6,7].

The advancing process of ageing is accompanied by quantitative and qualitative changes in human body cells that affect the ability to self-regeneration, differentiation, plasticity, and reacting to stimuli. Body composition changes with age, with increasing contents of body fat and decreasing contents of muscle mass, causing development of sarcopenic obesity. The muscle structure undergoes reorganization of motor units, changes of muscle spindle character and partial degeneration. This process limits motor activity, lowers effort tolerance, disturbs metabolism, and deepens the process of ageing [8].

Elderly people are affected by an increase in blood viscosity and increased concentrations of fibrinogen [9]. The changes result from the physiological process of body ageing, which weakens the mechanism of self-regulation of rheological blood parameters [10] connected with its flow through blood vessels. Depending on the conducted research, it is estimated that the physiological increase of fibrinogen concentration in blood plasma varies from 10 mg/dL to 13.6 mg/dL per a decade of human life. Increased concentrations of fibrinogen is also observed in obese people [11,12].

Ageing also causes a decrease in erythrocyte deformability [13]. The fall of erythrocyte deformability and increased aggregation of red blood cells are connected with the occurrence of diseases such as hypertension, diabetes, and coronary artery disease [14,15]. The factors favoring creation of blood cell aggregates comprise, among others, a higher level of fibrinogen concentration [16]. A decrease in the proliferative activity of stem cells with age also causes a decreased production of erythrocytes [17]. Therefore, ageing results in lowering hematological values in the body; the number of blood cellular components decreases, the level of hemoglobin lowers, and the mean corpuscular volume also falls [13].

Besides disorders connected with body composition, lowering hematological values, blood flow, and fibrinogen level, the body in the ageing phase can also be affected by disturbances of protein management. In elderly people the measurement of protein allows, among others, determining the nutritional condition and liver function, where it is synthesized [18]. This parameter is connected with the immune system, maintains endovascular osmotic pressure, and controls the transport of metabolites [19,20].

Elderly people need activities that will positively contribute to a satisfactory process of ageing; that is, getting old with a lower risk of becoming ill and maintaining mental and physical fitness [1]. Therefore, there have been continuous attempts to find an efficient program to activate the elderly and to promote a healthy lifestyle and physical activity, especially in this social group.

So far only a few studies have been conducted to determine how vibrotherapy, which induces TVR and influences the morphological and rheological parameters of blood and the level of basic proteins and fibrinogen concentrations in elderly people. Moreover, the research analyzing the influence of vibrotherapy differs regarding the methodology, applied parameters of vibration, and devices. Therefore, in the present work it was decided to assess the influence of thirty vibrotherapy treatments on morphological and rheological blood parameters, the level of basic proteins, i.e., albumin and globulin, as well as fibrinogen concentration in blood in males aged 60–70 years, and also to check how the therapy affects body fatness in the examined.

## 2. Materials and Methods

### 2.1. Materials

**Figure 1:**
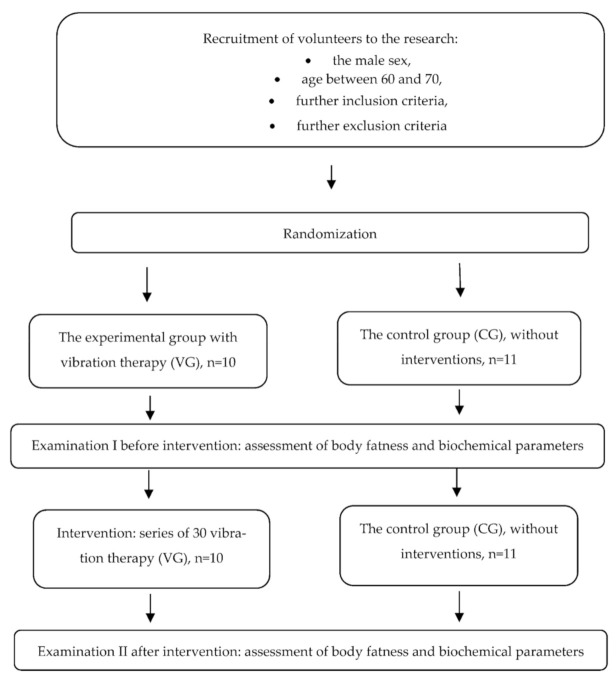
The course of the study.

The study design was a prospective, randomized, and controlled trial study to investigate the impact of cycloidal vibration therapy in elderly men and to estimate the change in participants’ body fatness; hematological, rheological, and protein blood parameters; and fibrinogen concentration. The course of the study is presented in Figure 1.

The study included 21 males aged 60–70 years (mean age 65.3 ± 2.7). The participants were assigned randomly to an experimental group and a control group; qualification was based on simple randomization following the order of applications. The experimental group received vibration therapy (VG), *n* = 10, whereas the other was the control group (CG), *n* = 11 without any interventions. The number of participants required to show statistical significance was based on previously published studies in this field. An error probability (α) of 0.05, power (1 − β) of 0.80, and an average effect size (d) of 0.8 were used to calculate the sample size.

The study inclusion criteria were male sex, age between 60 and 70, written consent to take part in the examinations, and lack of contraindications for undertaking physical activity.

The exclusion criteria were lack of consent to participate in examinations; motion limitations that made vibrotherapy impossible; advanced illnesses of blood vessels (aneurysm, thrombosis, atherosclerosis); conditions of recent heart attacks and strokes; conditions after bone fractures till complete adhesion; conditions after breaking tendons, ligaments, and muscles till fully regenerated; and after other treatments: endoprosthesis, implantation, reconstruction, and other surgeries till completely healed; acute infections caused by bacteria, fungi, or viruses, including skin infections; abscesses; non-controlled hypertension; advanced renal calculi and gallstones; acute condition of multiple sclerosis; epilepsy; illnesses with vertigo; insufficient mental fitness; syringomyelia; hemorrhage; increased body temperature; or active cancer.

The volunteers were informed about the procedures and purpose of the research in detail and about the possibility of resigning from participation at any stage without giving reasons. During the period of using vibrotherapy there were no resignations from the study, including for organizational or health reasons. All the examined were asked not to alter their nutritional habits, taken medicines, or level of physical activity during the experiment. All volunteers provided written consent for participation in the study, as well as for the use of personal data and research results for scientific purposes. The research project obtained the approval of the Ethics Committee of the Regional Medical Chamber in Krakow 3/KBL/OIL/2019.

### 2.2. Methods

For the purpose of this study body height (Ht) (cm) and body mass (BM) (kg) were used. Height was measured without shoes, in a standing position to the nearest 1 mm, with the head in the Frankfurt plane, using an anthropometer. Weight was obtained in the standing position with a standardized medical scale, with an accuracy of 100 g. Body mass index (BMI) (kg/m^2^) was computed according to the following formula: BMI = weight (kg)/height^2^ (m^2^). Body composition assessment: fat free mass (FFM) (kg), body fat mass (BF) (kg), percentage of body fat (BF) (%), and total body water (TBW) (%) were recorded using a Tanita analyzer (Tanita BC-601 Tanita Corporation, Tokyo, Japan), according to the manufacturer’s guidelines.

Fasting blood samples were collected in the morning from the basilic, cephalic, or median cubital vein into test tubes, with EDTA for hematological analysis of whole blood; K_2_ potassium edetate (6 mL) was used as an anticoagulant; with clotting activator, for plasma testing; the main activator ingredient was SiO_2_ (6 mL). The blood was collected by a qualified laboratory diagnostician, under the supervision of a physician, in accordance with the applicable standards of the Blood Physiology Laboratory of the University of Physical Education in Krakow.

The following hematological blood parameters were assessed with an ABX Micros 60 hematology analyzer (USA): hemoglobin (Hb) (g/dL), hematocrit (Hct) (%), red blood cells (RBC) (T/L), white blood cells (WBC) (10^9^/L), platelets (PLT) (10^9^/L), mean platelet volume (MPV) (fl), platelet distribution width (PDW) (%), plateletcrit (PCT) (%), mean corpuscular volume (MCV) (fl), mean corpuscular hemoglobin (MCH) (pg), mean corpuscular hemoglobin concentration (MCHC) (g/dL), red blood cell distribution width (RDW-CV) (%), and red blood cell distribution width, standard deviation (RDW-SD) (fl).

A laser-optical rotational cell analyzer (LORCA) (RR Mechatronics, the Netherlands) was used to study erythrocyte aggregation, and the results were presented as aggregation indices. The tests in the above-mentioned device were performed within 30 min of blood collection, at 37 °C, and in accordance with a standard protocol [21]. Parameters determining the kinetics of erythrocyte aggregation were investigated: aggregation index (AI) (%), total extent of aggregation (AMP, amplitude) (a.u.), half time kinetics of aggregation (T½) (s). To obtain information about the aggregation of red blood cells, 1–2 mL of a blood sample was transferred into a glass vessel and oxygenated with ambient air for 10 min. Then, 1 mL of oxygenated blood was added to the cup of a LORCA analyzer. The cup was set into rotational movement within 120 s. The blood sample was sheared at 400 s^−1^ and after 10 s, centrifugation was promptly stopped and red blood cells would begin to aggregate. The aggregation index was calculated using a syllectogram. Afterwards, the aggregation index, T½, and AMP were calculated using a computer program.

The fibrinogen (Fib) (g/L) concentration assessments were performed with a Bio-Ksel Chrom-7 camera (spectrophotometric method). The measurement was based on changes in optic density occurring during the clotting reaction and kinetic analysis of the process.

In the plasma obtained from collected blood the proteins were marked (g/L) (total proteins, albumins, α-1-globulins, α-2-globulins, β-1-globulins, β-2-globulins, γ-globulins). Total plasma protein was measured by using a Cobas 6000 analyzer, Roche and a Proteinogram-Minicap Sebia analyzer (biuret method).

### 2.3. Description of the Intervention

The vibrotherapy group (VG) took part in interventions on mattresses generating oscillatory-cycloid vibrations. The patients received vibrotherapy treatments in the morning, once a day, five times a week. They were obligated to participate in three courses, and one course comprised ten vibration treatments and ten days of resting. One vibration treatment lasted 29 min and included 8 microprograms. The treatments were performed in a prone position with the use of a RAM Vitberg+ Base Module (active medical device, class IIa) enhanced with a RAM Vitberg+ Metabolism module (active medical device, class I) [22]. The whole RAM Vitberg+ system combined a general action following the WBV rule (whole body vibration: oscillations of a general character) with a local action, applying an additional therapeutical module in which the vibration impulse of the RAM Vitberg+ Metabolism was directed towards the areas: epigastrium, mid-abdomen, pubic region, inguinal region, lateral and hypochondriac regions of abdomen, superficial fascia, and the layer between subcutaneous tissue and abdominal muscles. The indexes of the applied vibration changed over time according to the characteristics of the program. The therapeutic stimulus consisted of cycloid vibrations, produced in three perpendicular directions (3D), inducing intermittent pulsations with variable values of frequency (f), amplitude (A), and acceleration (a), which ranged between 25–52 Hz, 0.1–0.5 mm, and 6.9–13.5 m/s^2^, respectively. All of the trainings were carried out at the same time of day (in the morning) by the same physiotherapist, in a room with the same temperature and humidity.

### 2.4. Statistical Analysis

The distribution of results for the analyzed variables was checked by applying a Shapiro–Wilk test. For single measurements, the significance of group-related differences was assessed using independent-sample tests, Student’s *t*-test or the Mann–Whitney U test. In the VG and CG, the values of differences (∆) were assessed between the first and second examinations and the level of statistical significance was estimated with the pairs order Wilcoxon test. For comparing the impact of vibration treatments on changes in the analyzed variables among the compared groups, analysis of variance with repeated measures (ANOVA) was used, examining the influence of the main factors, i.e., group (vibration and control), treatment (influence of vibration therapy) and group × treatment interaction for variables of normal distribution. The statistical significance of differences was assumed for a level of *p* < 0.05. The STATISTICA 13 package (StatSoft, Inc., Tulsa, OK, USA) was used for calculations.

## 3. Results

The examined group (VG) included 10 men of mean age 65.6 ± 3.0 years, who received 30 vibrotherapy treatments over three months. The control group (CG) consisted of 11 men of mean age 65.0 ± 2.4 years. There were no differences between CG and VG with respect to BM, BMI, FFM, FM, BF%, and TBW before the interventions (Table 1).

Furthermore, for the majority of the remaining initial parameters, i.e., blood morphology (besides MCV and RDW-SD), in indexes of erythrocytes aggregation, and in proteinogram and the level of fibrinogen no significant differences were indicated between CG and VG before the interventions (Tables S1–S3, Supplementary Materials).

After applying three courses of vibrotherapy in the examined men (VG), a significant decrease was confirmed in the following body composition parameters: BM (∆BM: −2.7 ± 2.0; *p* = 0.002), BMI (∆BMI: −0.9 ± 0.7; *p* = 0.002), BF (∆BF: −2.5 ± 2.5; *p* = 0.013), and %BF (∆%BF: −2.0 ± 2.7; *p* = 0.041), between the first and second examinations (Table 2). In CG no changes in the analyzed parameters were found after three months (Table 2). The results of variance analysis confirmed changes over time of (BM: f = 12.585, *p* = 0.002; BMI: f = 13.041, *p* = 0.002; BF: f = 11.248, *p* = 0.003; %BF: f = 7.760, *p* = 0.012), though they did not indicate interactions between the groups (Table 2).

After the applied interventions, no changes were found in the level of most parameters of blood morphology; however, for Hb (∆ Hb: −0.4 ± 0.5; *p* = 0.047), RBC (∆ RBC: −0.1 ± 0.1; *p* = 0.035), and MCH (∆ MCH: −0.4 ± 0.4; *p* = 0.013) the parameter values slightly lowered in the VG. This tendency was confirmed for changes in time for RBC (f = 5.686, *p* = 0.021), but an interaction between the groups was not observed (Table 3).

After three months, the changes in both indexes of erythrocytes aggregation and in proteinogram were not confirmed, either in the inner or outer group analyses (Table 4 and Table 5).

**Figure 2:**
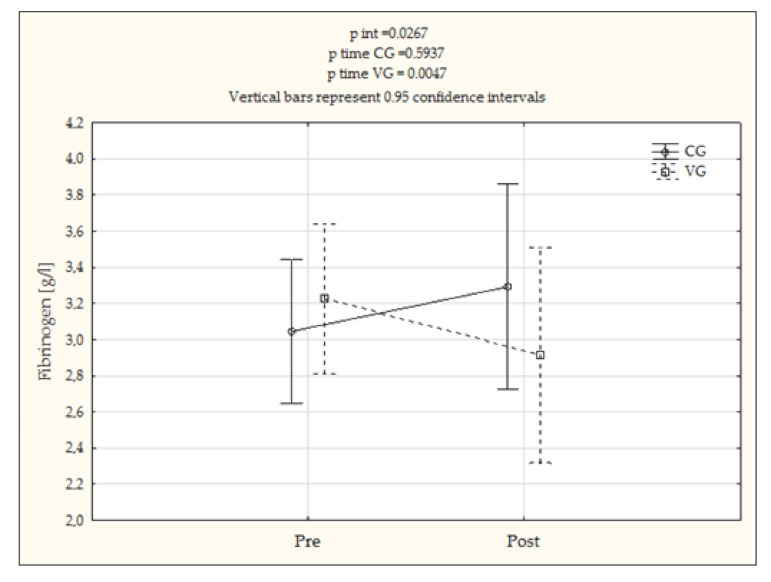
Mean concentrations of fibrinogen (g/L) in the control group (CG) and the examined group (VG) before (pre) and after (post) three-month-long vibrotherapy, considering interactions between groups (*p* int) and changes over time (*p* time).

It was demonstrated that after three courses of treatments a significant decrease in fibrinogen level took place (∆Fib: −0.3 ± 0.3, *p* = 0.005) in the examined men who received vibrotherapy (VG) (Figure 2). The occurrence of differences between groups was also confirmed (f = 5.770, *p* = 0.027). In the CG no significant changes in the analyzed parameters were found after three months (Table 4 and Table 5).

## 4. Discussion

The beneficial influence of vibration training, especially for the whole body, on weight, BMI and content of fat tissue in the body has been well presented in literature. It has been proven that vibration training of the whole body affects body weight reduction [23,24,25]. A higher decrease of BMI and fat mass in patients realizing vibration training of the whole body in comparison with those practicing aerobic training with diet control was also confirmed by researchers [26]. Rubin et al. [27] proved in their studies based on molecular techniques that vibration training may be an important non-pharmacological approach for obesity and its consequences. This theory was also confirmed by Vissers et al. [24], demonstrating that a combination of whole body vibration training, aerobic exercises, and low-caloric diet helps to achieve a significant body mass decrease (5–10%). Similar conclusions were drawn by Wilms et al. [26], who observed that energy expenditure during aerobic exercises combined with whole body vibration training was slightly higher if compared with the same training performed without vibration. The present research included a group of males of mean age 65.6 ± 3.0 years, who featured an increased level of BMI values and percentage fat content in the body. The presented research in the vibrotherapy group confirmed a significant decrease over time of the following body composition parameters: body weight, BMI, fatness, and percentage fat content in body, while no significant changes were observed in the control group.

An analysis of the literature confirmed the positive influence of BMI on fibrinogen concentration, which increases the aggregation of blood cells and plasma viscosity, and subsequently strengthens resistance in micro circulation [28,29,30]. Tripolino et al. [31] also indicated significant links between body weight and blood viscosity. The theory connecting the increase of fibrinogen concentration in blood with an increase of RBC aggregation was confirmed by Christy et al. [32]; while the study by Avellone et al. [33] demonstrated a correlation between plasma viscosity and fibrinogen concentration. Physical activity is believed to be, among others, a factor affecting decrease of fibrinogen concentration, especially in elderly people [34]. People undertaking regular physical activity are characterized by a more efficient fibrinolytic processes and lowered risk of clots [35,36] in comparison with inactive patients. In the study of Straton et al. [37], in a group of males aged 60–80 years who performed a regular six-month-long training, a decrease of fibrinogen by 13% was observed. Simmonds et al. [30] demonstrated the influence of regular physical effort on decreasing fibrinogen concentration in plasma, followed by changes in plasma viscosity and in whole blood properties. Whereas, Ikeda et al. [38] observed an increase in blood viscosity after physical effort, disregarding the duration of the activity and whether it was submaximal or maximal effort. It was shown that the changes correlated both with an increase of hematocrit level and plasma viscosity. This can be connected with the phenomenon of hemoconcentration that occurs after effort of a submaximal and maximal character, disregarding duration (short-term and long-term efforts) in the first phase after completing them, and that is affected by, among others, an increase of the number of red blood cells caused by spleen contraction, water loss in the thermoregulation process, and water transfer to muscle cells [39].

Vibration training, as a form of physical activity, can also affect the fibrinogen concentration and rheological parameters of blood [40]. Mechanical stimulation affects the functions of vessels, lowering the tension and stiffness of arteries [41]. Moreover, Rittweger et al. [42] mentioned a positive influence of vibration on peripheral circulation: oscillations extend blood vessels, blood perfusion through tissues increases, and muscles are better provided with oxygen and nutrients. Kerschan-Schindl et al. [4] demonstrated that vibrations can lower blood viscosity, and thus increase the average flow velocity. According to Ghazalian [43], vibration training indirectly affects the level of fibrinogen concentration through enhancing the function of endothelium. Vibrations generate pulsating shear forces acting on the endothelium, causing increased blood flow and higher activity of endothelial nitric oxide synthase (eNOS) and concentrations of nitric oxide (NO) [44]. The results of our research indicate a significant decrease of average concentration of fibrinogen, by 0.3 (g/L), in the examined vibrotherapy group, while in the control group the increase was 0.3 (g/L). The obtained results are particularly important, as the level of the protein is an independent cardiovascular risk factor, and there is a continuous search for methods that could lower its concentration in the body. No significant changes were found for indexes of blood aggregation, but in the vibrotherapy group there was a decreasing tendency in the aggregation indexation, which gains particular importance in light of theories connecting a high level of fibrinogen with the process of red blood cell aggregation, which may be favorable for, among others, micro circulation impairment, cell hypoxia, or clots. Lengthening of the exposition time could result in a stronger reaction from the rheological system; however, the applied three-month-long vibrotherapy program did not result in pathophysiological alterations to the rheological properties of blood.

In the research shown in the present work the analysis of blood red cell indexes indicates a lowering of the number of erythrocytes in males from the vibrotherapy group, which might be connected with an increase of plasma volume (PV). However, in the CG the mean growth of PV was 0.4%, whereas in the VG it was 2.8%; but the differences were not significant statistically (*p* = 0.490) (results not included). The increase of PV could be caused by adaptation to physical effort, when water loss takes place to keep the constant temperature of the body and then the water is restored, which also increases the plasma volume. Post-exercise hypervolemia is a kind of supercompensation that increases the activity of forces determining water content in the body after it was lost, trying to effectively compensate with a surplus the losses of plasma volume at the same time. A few factors may also affect the increase of plasma volume during exercise, e.g., an increase of metabolic production of water, release of water from glycogen complexes, water redistribution, changes in arterial pressure, increased flow of proteins, or change of body position [45]. Physical effort is a factor inducing oxidative stress, which induces vascular hemolysis and being the base for erythrocytes deterioration, including high density erythrocytes. The decrease in the membrane elasticity, as well as the reduced osmotic and mechanical resistance of erythrocytes, eventually lead to membrane fragmentation, increased rupturing of older erythrocytes exposed to mechanical factors, and changes that occur in the peripheral blood during physical stress [46]. Generally, individuals undertaking physical exercise feature a lower concentration of hemoglobin and a lower level of hematocrit, as well as reduced plasma and blood viscosity and greater elasticity in corpuscles, in comparison with individuals who lead a sedentary lifestyle. It is considered that the phenomenon of hemodilution lies at the basis of the above changes [47]. Results of the tests conducted in vitro indicate that vibrations can cause damages within erythrocytes; the higher the peak acceleration and the longer the exposition time for vibrations, the greater the damages to red blood cells [48]. This issue needs a deeper investigation, as there is lack of reports in the literature on the assessment of the influence of vibration training on the structure of red cells in elderly people, and the obtained results for changes over time were not confirmed by the level of interaction between the analyzed groups.

The total level of protein in blood depends on an equilibrium between production and disintegration of the two main protein fractions of albumin and globulin. Reports concerning the influence of physical activity on levels of protein in blood plasma show that the frequency of the performed aerobic exercises increases the level of albumin, whereas endurance training decreases the level of globulin [49]. Higher levels of albumin in active individuals may be an adaptation to exercise training to expand plasma volume [50]. In the examined vibrotherapy group there were no significant changes in the protein indexes; therefore the applied vibrotherapy program did not lead to pathophysiological changes, and the equilibrium between production and disintegration of the main protein fractions was maintained.

## 5. Limitations

A major limitation of this study was the small number of participants. Another issue was connected with the lack of measurement of anti- and pro-coagulatory markers [51] and comparison of the vibrotherapy efficiency between sexes [52]. The potential for observer bias is also acknowledged, as the researcher was not blinded and had prior knowledge of the research aims, disease status, and intervention. As such, these could all have influenced data recording. The researcher tried to minimize the risk of bias by following a standardized protocol for enrolment. The potential of reporting bias and observer bias could be reduced by implementing blinding in future studies.

## 6. Conclusions

In conclusion, it should be emphasized that the three-month-long vibrotherapy program positively influenced the mean concentration of fibrinogen in elderly males and resulted in lowering the content of fat tissue in the body, body weight, and BMI. The obtained results are particularly important, as the level of the fibrinogen is an independent cardiovascular risk factor and there is a continuous search for therapeutical methods that could lower its concentration in the body. This research has highlighted a number of issues that warrant future research. Further research should examine the effectiveness of vibrotherapy, ideally in a multi-center double-blind randomized controlled trial design, potentially using a placebo dummy machine, and on a greater number of patients. This should include a health economic evaluation, which could be compared to current treatment options. This would provide valuable information about the translation and transition of vibrotherapy into everyday healthcare.

## Figures and Tables

**Table 1:** Values of anthropometric parameters in the whole group and in the control (CG) and the examined (VG) groups before applying vibrotherapy.

Variables	Total	CG	VG	*p*
	X ± SD	X ± SD	X ± SD	
Age	65.3 ± 2.7	65.0 ± 2.4	65.6 ± 3.0	0.619
BM (kg)	85.6 ± 8.2	84.2 ± 6.7	87.2 ± 9.6	0.412
BMI (kg/m^2^)	27.8 ± 3.4	26.5 ± 2.7	29.3 ± 3.3	0.053
FFM (kg)	53.2 ± 4.2	53.8 ± 4.1	52.5 ± 4.5	0.499
BF (kg)	29.7 ± 8.9	27.5 ± 8.2	32.1 ± 9.4	0.251
BF (%)	34.2 ± 7.6	32.3 ± 7.9	36.2 ± 7.0	0.235
TBW (%)	50.8 ± 4.6	51.5 ± 4.2	50.0 ± 5.2	0.490

X—mean, SD—standard deviation, BM—Body Mass, BMI—Body Mass Index, FFM- Fat Free Mass, BF—Body Fat, TBW—Total Body Water.

**Table 2:** Values of anthropometric parameters and their differences (∆) in the control (CG) and examined (VG) groups before and after three vibrotherapy series, considering interactions between the groups (*p* int) and changes over time (*p* time).

Variables	CG				VG				*p* Int	*p* Time
	Pre	Post	∆	*p*	Pre	Post	∆	*p*		
	X ± SD	X ± SD	X ± SD		X ± SD	X ± SD	X ± SD			
BM (kg)	84.2 ± 6.7	83.1 ± 6.0	−1.1 ± 2.8	0.225	87.2 ± 9.6	84.5 ± 9.9	−2.7 ± 2.0	0.002 *	0.150	0.002 *
BMI (kg/m^2^)	26.5 ± 2.7	26.2 ± 2.4	−0.4 ± 0.9	0.218	29.3 ± 3.3	28.4 ± 3.4	−0.9 ± 0.7	0.002 *	0.136	0.002 *
FFM (kg)	53.8 ± 4.1	53.6 ± 3.6	−0.2 ± 1.3	0.660	52.5 ± 4.5	51.9 ± 3.8	−0.6 ± 1.6	0.245	0.486	0.218
BF (kg)	27.5 ± 8.2	26.6 ± 7.7	−0.9 ± 2.1	0.181	32.1 ± 9.4	29.6 ± 10.4	−2.5 ± 2.5	0.013 *	0.131	0.003 *
BF (%)	32.3 ± 7.9	31.7 ± 7.5	−0.6 ± 1.5	0.222	36.2 ± 7.0	34.2 ± 8.6	−2.0 ± 2.7	0.041 *	0.149	0.012 *
TBW (%)	51.5 ± 4.2	51.9 ± 4.1	0.4 ± 1.1	0.288	50.0 ± 5.2	50.4 ± 4.6	0.4 ± 1.2	0.380	0.922	0.152

X—mean, SD—standard deviation, *p*—* statistically significant value (*p* < 0.05), BM—Body Mass, BMI—Body Mass Index, FFM—Fat Free Mass, BF—Body Fat, TBW—Total Body Water.

**Table 3:** Values of blood morphological parameters and their differences (∆) in the control (CG) and examined (VG) groups, before and after three vibrotherapy series considering interactions between the groups (*p* int) and changes over time (*p* time).

Variables	CG				VG				*p* Int	*p* Time
	Pre	Post	∆		Pre	Post	∆			
	X ± SD	X ± SD	X ± SD	*p*	X ± SD	X ± SD	X ± SD	*p*		
Hb (g/dL)	14.9 ± 0.7	14.7 ± 0.8	−0.2 ± 0.7	0.515	15.3 ± 0.6	14.9 ± 0.4	−0.4 ± 0.5	0.047 *	0.438	0.078
Hct (%)	43.7 ± 2.6	43.7 ± 2.4	−0.0 ± 2.1	0.978	44.4 ± 1.7	43.7 ± 1.0	−0.7 ± 1.4	0.180	0.439	0.414
RBC (T/L)	4.8 ± 0.4	4.7 ± 0.3	−0.2 ± 0.3	0.121	5.0 ± 0.3	5.0 ± 0.2	−0.1 ± 0.1	0.035 *	0.517	0.021 *
WBC (10^9^/L)	5.6 ± 1.2	5.9 ± 1.1	0.4 ± 1.2	0.332	6.0 ± 0.7	6.0 ± 0.5	0.0 ± 0.5	0.902	0.413	0.601
PLT (10^9^/L)	220.1 ± 63.2	216.1 ± 32.4	−4.0 ± 50.9	0.563	225.6 ± 50.5	219.8 ± 57.2	−5.8 ± 20.6	0.203	0.918	0.808
MPV (fl)	12.1 ± 0.7	12.6 ± 0.2	−0.0 ± 0.6	0.424	12.0 ± 1.0	12.1 ± 1.1	0.1 ± 0.7	0.779	0.783	0.883
PDW (%)	15.8 ± 0.2	15.9 ± 0.0	0.1 ± 0.2	0.240	15.8 ± 0.2	15.9 ± 0.2	0.1 ± 0.2	0.191	0.930	0.077
PCT (%)	0.3 ± 0.1	0.3 ± 3.4	−0.0 ± 0.1	0.657	0.3 ± 0.1	0.3 ± 0.1	−0.0 ± 0.0	0.418	0.893	0.695
MCV (fl)	91.2 ± 3.2	91.5 ± 1.2	0.3 ± 1.0	0.351	88.4 ± 2.2	88.0 ± 2.0	−0.3 ± 0.5	0.060	0.080	0.578
MCH (pg)	30.9 ± 1.1	30.9 ± 0.8	0.0 ± 0.7	0.862	30.3 ± 1.0	29.9 ± 1.1	−0.4 ± 0.4	0.013 *	0.099	0.167
MCHC (g/dL)	33.9 ± 0.8	33.8 ± 10.5	−0.1 ± 0.6	0.523	34.3 ± 0.6	34.0 ± 0.8	−0.3 ± 0.6	0.151	0.550	0.136
RDW-CV (%)	13.8 ± 0.5	17.1 ± 2.0	3.4 ± 10.5	0.110	13.6 ± 0.5	13.8 ± 0.4	0.2 ± 0.3	0.101	0.349	0.676
RDW-SD (fl)	46.4 ± 2.4	46.5 ± 0.2	0.1 ± 1.5	0.827	44.2 ± 1.7	44.5 ± 1.4	0.4 ± 1.1	0.346	0.660	0.439

X—mean, SD—standard deviation, *p*—* statistically significant value (*p* < 0.05), Hb—Hemoglobin, Hct—Hematocrit, RBC—Red Blood Cells, WBC—White Blood Cells, PLT—Platelets, MPV—Mean Platelet Volume, PDW—Platelet Distribution Width, PCT—Plateletcrit, MCV—Mean Corpuscular Volume, MCH—Mean Corpuscular Hemoglobin, MCHC—Mean Corpuscular Hemoglobin Concentration, RDW-CV—Red Blood Cell Distribution Width, RDW-SD—Red Blood Cell Distribution Width-Standard Deviation.

**Table 4:** Values of indexes of erythrocytes aggregation and fibrinogen concentration and their differences (∆) in the control (CG) and examined (VG) groups before and after three vibrotherapy series, considering interactions between the groups (*p* int) and changes over time (*p* time).

Variables	CG				VG				*p* Int	*p* Time
	Pre	Post	∆		Pre	Post	∆			
	X ± SD	X ± SD	X ± SD	*p*	X ± SD	X ± SD	X ± SD	*p*		
AMP (au)	21.0 ± 3.3	21.7 ± 2.7	0.7 ± 2.8	0.466	23.5 ± 2.0	23.6 ± 1.8	0.1 ± 2.8	0.921	0.656	0.556
T½ (s)	2.1 ± 0.7	1.8 ± 0.4	−0.3 ± 0.7	0.184	2.0 ± 0.5	1.8 ± 0.4	−0.1 ± 0.6	0.487	0.573	0.141
AI (%)	65.7 ± 8.1	67.4 ± 7.5	1.8 ± 7.1	0.328	63.6 ± 5.2	61.9 ± 4.5	−1.7 ± 5.1	0.323	0.222	0.520
Fib (g/L)	3.1 ± 0.6	3.3 ± 1.1	0.3 ± 0.7	0.594	3.2 ± 0.7	2.9 ± 0.5	−0.3 ± 0.3	0.005 *	0.027 *	0.192

X—mean, SD—standard deviation, *p*—* statistically significant value (*p* < 0.05), AMP—amplitude of aggregation, T½—half time kinetics of aggregation, AI—aggregation index, Fib—fibrinogen.

**Table 5:** Values of the protein profile parameters and their differences (∆) in the control (CG) and examined (VG) groups before and after three vibrotherapy series, considering interactions between the groups (*p* int) and changes over time (*p* time).

Variables	CG				VG				*p* Int	*p* Time
	Pre	Post	∆		Pre	Post	∆			
	X ± SD	X ± SD	X ± SD	*p*	X ± SD	X ± SD	X ± SD	*p*		
Total proteins (g/L)	74.7 ± 4.5	72.1 ± 2.7	−2.7 ± 5.2	0.182	73.5 ± 3.8	72.5 ± 3.7	−1.0 ± 1.9	0.143	0.347	0.085
Albumins (g/L)	58.3 ± 4.5	58.4 ± 4.4	0.1 ± 4.1	0.972	58.7 ± 3.3	57.2 ± 9.5	−1.4 ± 4.5	0.169	0.658	0.313
α-1-globulins (g/L)	3.1 ± 3.2	2.5 ± 0.8	−0.7 ± 3.3	0.721	2.2 ± 0.3	2.1 ± 0.3	−0.1 ± 0.3	0.069	0.626	0.514
α-2-globulins (g/L)	10.8 ± 1.4	11.2 ± 22.2	0.4 ± 1.8	0.542	10.3 ± 0.9	9.8 ± 1.3	−0.5 ± 1.0	0.140	0.237	0.414
β-1-globulins (g/L)	9.0 ± 1.1	9.0 ± 0.5	0.0 ± 0.9	1.000	9.4 ± 0.8	9.5 ± 1.0	0.1 ± 0.7	0.686	0.784	0.807
β-2-globulins (g/L)	7.4 ± 1.4	7.1 ± 1.4	−0.3 ± 0.9	0.610	6.9 ± 1.6	6.6 ± 1.4	−0.3 ± 0.7	0.198	0.855	0.185
γ-globulins (g/L)	12.4 ± 2.3	10.7 ± 2.7	−1.7 ± 3.2	0.091	12.4 ± 2.1	12.3 ± 1.7	−0,1 ± 1.2	0.851	0.146	0.092

X—mean, SD—standard deviation.

## Supplementary Materials

The following are available online at https://www.mdpi.com/article/10.3390/jcm10153259/s1, Table S1. Values of blood morphological parameters in the whole group and in the control (CG) and the examined (VG) ones before applying vibrotherapy, Table S2. Values of indexes of erythrocytes aggregation and fibrinogen concentration in the whole group and in the control (CG) and the examined (VG) ones before applying vibrotherapy, Table S3. Values of the protein profile parameters in the whole group and in the control (CG) and the examined (VG) ones before applying vibrotherapy.
