# Supplementary material for: Effect of Vibrotherapy on Body Fatness, Blood Parameters and Fibrinogen Concentration in Elderly Men
Source: J Clin Med. 2021 Jul 23;10(15):3259. doi: 10.3390/jcm10153259 (PMC8347586; doi:10.3390/jcm10153259)
Supplement: Supplementary file 1 [file jcm-10-03259-s001.zip › jcm-1306751-supplementary.pdf]

Table S1. Values of blood morphological parameters in the whole group and in the control (CG) and the examined (VG) ones before applying vibrotherapy.

Variables	Total	CG	VG	*p*
	X ± SD	X ± SD	X ± SD	
Hb [g/dl]	15.0 ± 0.7	14.9 ± 0.7	15.3 ± 0.6	0.163
Hct [%]	44.0 ± 2.2	43.7 ± 2.6	44.4 ± 1.7	0.509
RBC [T/L]	4.9 ± 0.4	4.8 ± 0.4	5.0 ± 0.3	0.223
WBC [10^9/L]	5.8 ± 1.0	5.6 ± 1.2	6.0 ± 0.7	0.159
PLT [10^9/L]	222.7 ± 56.2	220.1 ± 63.2	225.6 ± 50.5	0.829
MPV [fl]	12.1 ± 0.9	12.1 ± 0.7	12.0 ± 1.0	0.912
PDW [%]	15.8 ± 0.2	15.8 ± 0.2	15.8 ± 0.2	0.972
PCT [%]	0.3 ± 0.1	0.3 ± 0.1	0.3 ± 0.1	0.917
MCV [fl]	89.8 ± 3.0	91.2 ± 3.2	88.4 ± 2.2	0.030*
MCH [pg]	30.6 ± 1.1	30.9 ± 1.1	30.3 ± 1.0	0.232
MCHC [g/dl]	34.1 ± 0.7	33.9 ± 0.8	34.3 ± 0.6	0.248
RDW-CV [%]	13.7 ± 0.5	13.8 ± 0.5	13.6 ± 0.5	0.389
RDW-SD [fl]	45.3 ± 2.3	46.4 ± 2.4	44.2 ± 1.7	0.026*

X – mean, SD – standard deviation, p – * statistically significant value ($p<0.05$), Hb – hemoglobin, Hct – hematocrit, RBC – Red Blood Cells, WBC – White Blood Cells, PLT – Platelets, MPV- Mean Platelet Volume, PDW – Platelet Distribution Width, PCT– Plateletcrit, MCV - Mean Corpuscular Volume, MCH - Mean Corpuscular Hemoglobin, MCHC - Mean Corpuscular Hemoglobin Concentration, RDW-CV Red blood cell Distribution Width, RDW-SD – Red blood cell Distribution Width-Standard Deviation.

Table S2. Values of indexes of erythrocytes aggregation and fibrinogen concentration in the whole group and in the control (CG) and the examined (VG) ones before applying vibrotherapy.

Variables	Total	CG	VG	*p*
	**X ± SD**	**X ± SD**	**X ± SD**	
AMP [au]	22.2 ± 3.0	21.0 ± 3.3	23.5 ± 2.0	0.051
T ½ [s]	2.0 ± 0.6	2.1 ± 0.7	2.0 ± 0.5	0.747
AI [%]	64.7 ± 6.8	65.7 ± 8.1	63.6 ± 5.2	0.504
Fib [g/l]	3.1 ± 0.6	3.1 ± 0.6	3.2 ± 0.7	0.522

X – mean, SD – standard deviation, AMP – Amplitude of aggregation, T½ – Half Time kinetics of aggregation, AI – Aggregation Index, Fib – fibrinogen.

Table S3. Values of the protein profile parameters in the whole group and in the control (CG) and the examined (VG) ones before applying vibrotherapy.

**Variables**	**Total**	**CG**	**VG**	***p***
	**X ± SD**	**X ± SD**	**X ± SD**	
Total proteins [g/l]	74.1 ± 4.2	74.7 ± 4.5	73.5 ± 3.8	0.520
Albumins [g/l]	58.5 ± 3.9	58.3 ± 4.5	58.7 ± 3.3	0.833
α-1-globulins [g/l]	2.7 ± 2.3	3.1 ± 3.2	2.2 ± 0.3	0.888
α-2-globulins [g/l]	10.5 ±1.2	10.8 ± 1.4	10.3 ± 0.9	0.315
β-1-globulins [g/l]	9.2 ± 1.0	9.0 ± 1.1	9.4 ± 0.8	0.360
β-2-globulins [g/l]	7.2 ± 1.5	7.4 ± 1.4	6.9 ± 1.6	0.486
γ-globulins [g/l]	12.4 ± 2.2	12.4 ± 2.3	12.4 ± 2.1	0.999

X – mean, SD – standard deviation.
